# Study on Forming Law and Penetration of a Spherical Cone Composite Structure Liner Based on the Explosion Pressure-Coupling Constraint Principle

**DOI:** 10.3390/ma15144750

**Published:** 2022-07-07

**Authors:** Jilong Han, Zhonghua Du, Chao Zheng, Yongxu Wang, Yuqing Shang, Weiming Huang, Xi Wang, Jinbei Zhao

**Affiliations:** 1 First Research Institute, Ningbo Branch of China Academy of Ordnance Sciences, Ningbo 315103, China; qiugrass@126.com (C.Z.); wangyongxu216@163.com (Y.W.); diinm11@163.com (W.H.); cywangxi@163.com (X.W.); zhaojinbei1993@163.com (J.Z.); 2 School of Mechanical Engineering, Nanjing University of Science and Technology, Nanjing 210094, China; duzhonghua@aliyun.com; 3 Shanghai Electromechanical Engineering Research Institute, Shanghai 201109, China; 18260031785@163.com

**Keywords:** shaped charge, explosion pressure-coupling constraint principle, SCS liner, shaped charge jet

## Abstract

The liner is an important part of shaped charge. In this paper, the spherical cone composite structure liner composed of a spherical missing body and truncated cone (hereinafter referred to as the SCS liner) is studied. The SCS liner is made of copper. Based on this, a shaped charge structure based on the explosion pressure-coupling constraint principle is designed, filling an 8701 explosive (RDX-based explosive). Through pulse X-ray tests, numerical simulation, and static explosion tests, the significance of the detonation pressure-coupling constraint principle, as well as the forming law and penetration efficiency of the SCS liner are studied. The results show that in the pulsed X-ray test, a split jet with high velocity is formed in the SCS liner. The explosion pressure-coupling constraint principle delays the attenuation of the internal explosion pressure and improves the shape of jet. After the SCS liner is selected, the penetration depth is increased by 70.38%. The average head velocity of the explosive charge jet is 7594.81 m/s. The diameter of the hole formed by the jet of the explosive charge is 20.33 mm. The hole expands inside, and the perforation depth is 178.87 mm. The numerical simulation is in good agreement with the test.

## 1. Introduction

Shaped charge is a charge structure that uses the groove at one end of the explosive to concentrate energy [1,2,3,4]. Since the application of shaped charge, its structure has evolved and its performance has improved continuously. It has become an important anti-armor weapon [1]. The traditional single-cone, double-cone, horn-shaped liners, and hemispherical and spherical missing body liners are common structures, and a shaper is used to adjust the detonation waveform [2,3,4,5,6,7,8,9,10]. Small cone-shaped charge liners form a jet, large cone angle and hemispherical shapes form rod jets, and spherical missing body shaped charge liners form explosive-formed projectiles (EFP) [5,6,7,8]. The velocity and structure of jet are restricted by the material properties, charge structure, and liner shape [9]. The velocity of the traditional spherical missing body charge liner after forming is generally less than 3000 m/s, and the penetration depth is less than one times that of the charge diameter, which is difficult to break through [5,8]. Improving the velocity and penetration depth of the spherical missing body after forming is the focus of the current research.

In this paper, a composite structure charge containing coaxial internal and external charge columns is proposed. This charge structure uses the explosion pressure generated by the external charge column to limit the explosion pressure generated by the internal charge column, forming the coupling constraint principle of explosion pressure. In combination with the SCS gasket composed of a spherical missing body and a truncated cone, the two parts are made of copper. The spherical missing body material is expressed as “copper” and the truncated cone material is expressed as “copper-z” in the paper. The purpose is as follows: The spherical missing body forms the front of the jet and forms a large opening for the target plate. The truncated cone forms the rear of the jet, follows the front of the jet into the hole, and continues to penetrate the target plate or release fragments behind the target. Two groups of pulse X-ray tests, six groups of numerical simulation tests, and two groups of static explosion tests were carried out separately. The explosive pressure-coupling constraint principle is demonstrated for the first time. The forming mechanism and penetration performance of the charge structure are studied through pulse X-ray tests, numerical simulation, and static explosion tests.

## 2. Explosion Pressure-Coupling Constraint Principle and Action Process

**Figure 1:**
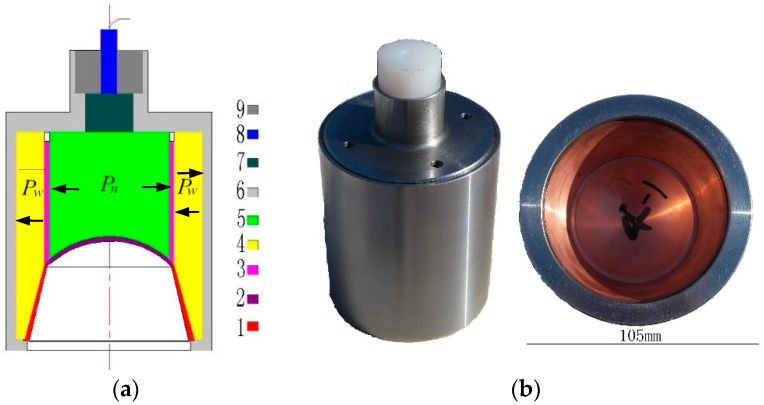
Schematic diagram of charge (**a**) and physical object of shaped charge based on the explosive pressure-coupling constraint principle (**b**). 1—Truncated cone; 2—spherical segment; 3—restraint cylinder; 4—external explosive column; 5—internal explosive column; 6—shell; 7—booster explosive; 8—detonator; 9—lightning tube socket.

**Figure 2:**
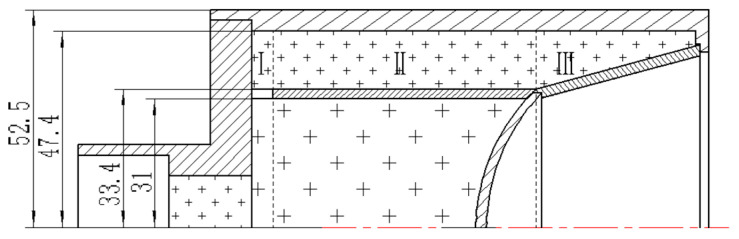
Schematic of the explosive pressure-coupling constraint principle. I—Impact initiation area; II—constraint area; III—ejection area.

We used an embedded constraint cylinder to separate the inner and outer explosive columns, as shown in Figure 1. The schematic diagram of the explosion pressure-coupling constraint principle is shown in Figure 2. The outer explosive column is divided into three zones: zone one is the impact initiation area, zone two is the constraint area, and zone three is the ejection area (mainly playing the role of ejecting the truncated cone, which is not analyzed here). After the initiation of the inner explosive column, the detonation wave propagates continuously forwards and outwards. When the detonation wave propagates to the inner surface of the outer explosive column in zone one, it is detonated rapidly, and then a stable detonation wave is generated in the outer grain. The pressure after the outer explosive column explodes (external explosive pressure, *P*_w_) will restrict the pressure of the internal explosion here (internal explosive pressure, *P*_n_), and the internal explosive pressure will also affect the external explosive pressure. This interaction leads to the coupling effect of the external and internal explosive pressures, increasing the peak value and work time of the internal explosive pressure [4]. This principle is called the explosive pressure-coupling constraint principle. The charging schematic diagram based on the explosive pressure-coupling constraint principle is shown in Figure 1.

Assuming the explosives detonate instantaneously, the initial detonation pressure is expressed by the average pressure for convenience of derivation, as shown in Equation (1) [10]:

(1) $$p_{c}=\rho_{0}D^{2}/8$$

where *ρ*_0_ is the initial density of explosive. With the progress of explosion, the radius *r* of the explosive gas increases continuously, resulting in continuous decrease in density *ρ*.

Since the mass of the product gas at the beginning is π*r*_0_^2^*lρ*_0_, the mass of the same gas at any other moment is π*r*^2^*lρ*. Here, *l* and *ρ* are the charge length of explosive and the immediate density of detonation products, respectively. *D* is the detonation velocity of the explosive, *r*_0_ is the initial radius of explosive, and *ρ*_0_ is the initial density of explosive. The relationship between the instantaneous detonation pressure and the immediate pressure of the expanded detonation product is shown in Equation (2) [10]:

(2) $$\left\{ \begin{array}{l} r^{2}\rho =r_{0}^{2}\rho_{0},p\propto \rho^{3}\\ \frac{p}{p_{c}}={\left(\frac{\rho }{\rho_{0}}\right)}^{3}={\left(\frac{r_{0}}{r}\right)}^{6} \end{array}\right.$$

Equation (3) can be obtained from Equation (2) [10]:

(3) $$\frac{p_{n}}{p_{cn}}=\frac{\rho_{n}^{3}}{\rho_{0}^{3}}={\left(\frac{r_{3}}{r_{3}^{\prime }}\right)}^{6}$$

The instantaneous average detonation pressure of an 8701 explosive is *p_c_* = 14.95 GPa [11]. When the internal detonation pressure expands to the surface of the outer explosive column (expanded to *r*_3_, $r_{4}^{\prime }$ = *r*_3_), the average immediate pressure of the internal detonation product decreases to *p*_1*n*_ = 9.55 GPa, which is greater than the initiation threshold of the 8701 explosive at 80 MPa [12]. The criterion equation of explosive impact initiation [13] is

(4) $$E_{c}=\frac{p_{cj}{}^{2}\tau }{\rho_{a}D_{a}}=p_{cj}u_{a}\tau$$

where *E_c_* is the shock wave energy acting on the unit area of explosive, *p_cj_* is the shock wave pressure, *u_a_* is the particle velocity on the array, *ρ_a_* is the density of the explosive to be fired, and *D_a_* is the velocity of the shock wave transmitted into the explosive to be fired. Therefore, according to the above equation, the outer explosive column detonates rapidly after Δ*τ* time (the time from ignition to stable detonation is very short).

In conclusion, it can be seen that

(5) $$\left\{ \begin{array}{l} p_{0w}=p_{c}\\ p_{0w}>p_{1n} \end{array}\right.$$

where *p*_0*w*_ is the instantaneous detonation pressure of the outer explosive column. The expansion of the detonation gas produced by the inner explosive column will be limited after the initiation of the outer explosive column. The detonation wave of the inner explosive column is reflected and superimposed on the axis to form a shock wave higher than the detonation pressure of Chapman–Jougue (CJ). As the detonation wave propagates along the axial direction, the detonation wave of the outer explosive column enters zone two, and the detonation wave of the outer explosive column is always slower than that of the inner explosive column by Δ*τ* time. In zone two, the constraint cylinder can transmit a shock wave, but the propagation speed of the shock wave in solids is less than the detonation speed of the outer explosive column. The shock wave passing through the constraint cylinder lags behind the detonation wave of the outer explosive column, which cannot make the outer explosive column impact ignition.

The air pressure is much lower than the explosion pressure of the inner explosive column, and the inward expansion of the explosive gas produced by the outer explosive column is much less than the expansion in the air. Therefore, without considering the inward expansion of the external explosion pressure, the internal and external pressures are equal after the outer explosive column expands for a certain time, which is a critical state, i.e.,

(6) $$p_{1n}^{\prime }=p_{1w}$$

where $p_{1n}^{\prime }$, *p*_1*w*_ are the explosion gas pressure of the inner and outer explosive columns at the equilibrium moment, respectively. According to Equation (2), the relationship between the external explosion pressure and the initial explosion pressure is

(7) $$\frac{p_{0w}}{p_{1w}}=\frac{\rho_{1w}^{3}}{\rho_{0}^{3}}=\left(\frac{r_{1}^{2}-r_{2}^{2}}{{\left(r_{1}^{\prime }\right)}^{2}-{\left(r_{2}^{\prime }\right)}^{2}}\right)$$

where *ρ*_1*w*_ is the density of the outer explosive column at the equilibrium moment. According to Equation (7), when the detonation product of the outer explosive column expands to $r^{\prime }$ = 50.97 mm, it reaches the state of Equation (6). The expansion limit of the cylindrical charge shell is 1.8 times the initial radius [10]. When the outer surface radius of the outer explosive column expands to 1.8 times the initial radius, the shell fails, and the sparse wave enters the detonation product of the outer explosive column, making its pressure decrease rapidly. At this moment, the outer surface of the inner explosive column also expands by 1.8 times. However, due to the constraint effect of internal and external detonation products, the constraint cylinder will not fail immediately, which can delay the propagation of the sparse wave to the detonation product of the inner explosive column, that is, delay the attenuation of the internal detonation pressure and increase the work time.

## 3. Numerical Simulation Study on Forming Law of SCS Liner

The two-dimensional axisymmetric model is established by using the nonlinear dynamics software Autodyn-2D. Firstly, the Euler method is used to establish the grid region, and the boundary condition of the grid region is set as outflow to simulate the forming of the SCS liner. Then, the formed jet properties are mapped to the Lagrangian grid to further solve the penetration of the jet [14]. The shell, confinement cylinder, and target column of the shaped charge are made of 45# steel (AISI/SAE standard no. 1045), the copper plate number used in the text is T2 (ASTM standard no. C11000), and the structure of the shaped charge is shown in Figure 1. The detonation gas of the inner explosive column is described by the JWL equation, and the detonation gas of the outer explosive column is described by the Lee Tarver equation. Lee Tarver equation is

(8) $$\frac{d\lambda }{dt}=I{\left(1-x\right)}^{b}{\left(\eta_{s}-1-a\right)}^{x}+G_{1}{\left(1-\lambda \right)}^{c}\lambda^{d}p^{y}+G_{2}{\left(1-\lambda \right)}^{e}\lambda^{g}P^{z}$$

where *λ* is the mass fraction of the reacted explosive, *η_s_* is the relative density of the impacted unreacted explosive, *P* is the local pressure (Mbar), and t is the time (μs), *I*, *a*, *b*, *x,* and *G*_1_, *c*, *d*, *y*, *G*_2_, *e*, *g*, *z* are constants.

The equation of state for impact is as follows

(9) $$u_{c}=C_{0}+Su_{p}$$

where *S* is a constant, *u_c_* is the shock wave velocity, *C*_0*c*_ is the sound velocity, and *u_p_* is the particle velocity. See Table 1 for material parameters.

### 3.1. Significance of Explosion Pressure Constraint Principle

**Figure 3:**
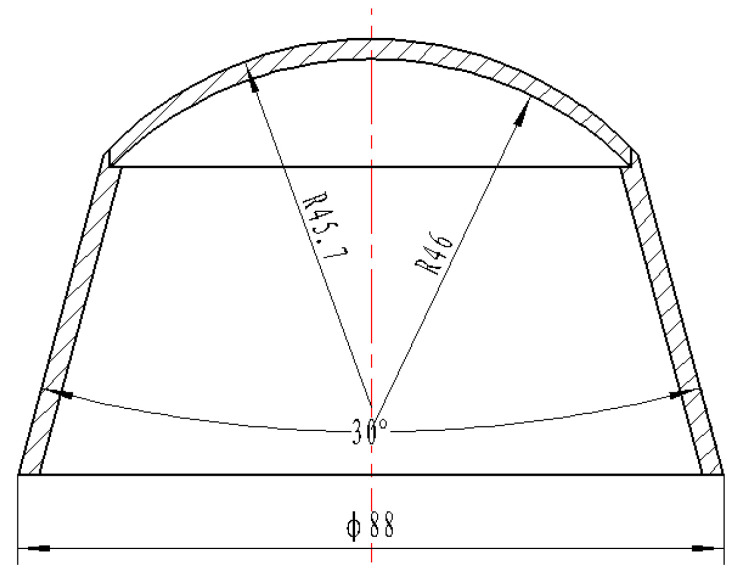
SCS liner.

**Figure 4:**
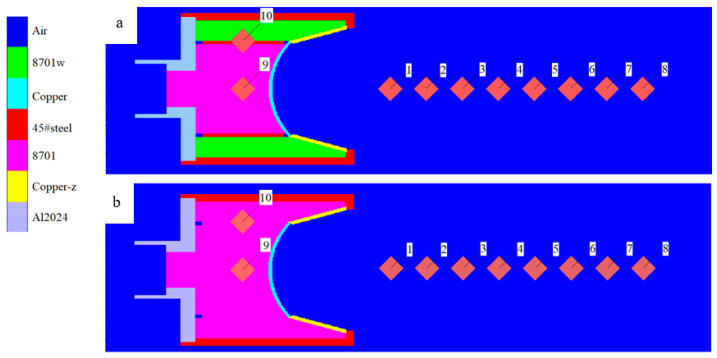
Numerical simulation model: (**a**) composite charge; (**b**) integrated charging.

By using the numerical simulation method, the significance of the explosion pressure-coupling constraint principle is analyzed. The structure of the SCS liner is shown in Figure 3, and the truncated cone is of equal wall thickness. Figure 4 is the numerical simulation model of the composite charge and integrated charge, with the structures of the liners the same as that in Figure 3. In Figure 4, Gauss points 1–8 are velocity-monitoring points, Gauss point 9 monitors the internal explosion pressure, and Gauss point 10 monitors the external explosion pressure.

**Figure 5:**
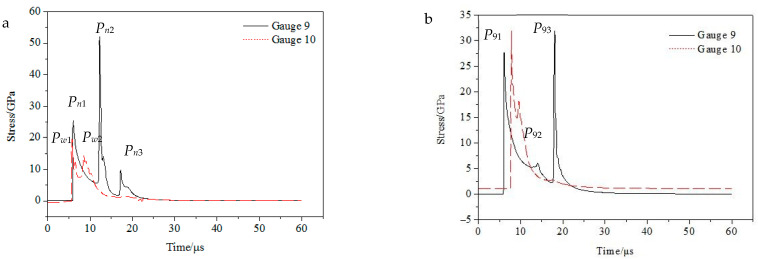
Burst pressure curve: (**a**) composite charge; (**b**) integrated charging.

In Figure 5, there are three peaks in each curve. The first peak is the CJ detonation pressure, and the second and third peaks are reflection peaks. As can be seen in Figure 5a, the external explosion pressure peak (*P_w_*_1_) is smaller than the internal explosion pressure peak (*P_n_*_1_), and the external reflection peak (*P_w_*_2_) occurs before the internal reflection peak (*P_n_*_2_), because the reflection distance of the external explosion pressure is smaller than that of the internal explosion pressure. In Figure 5a, due to the restriction of the external explosion pressure, the *P_n_*_2_ value reaches 50.90 GPa, which is significantly higher than the reflection peak (*P*_93_, 31.91 GPa) in Figure 5b; *P_n_*_2_ is about twice that of *P_n_*_1_, which is similar to the reflection of the rigid surface. The action time value higher than 7.5 GPa in Figure 5a is twice that of Figure 5b. At the internal explosion pressure *P_n_*_3_, the external explosion pressure also increases slightly, which shows the counteraction of the internal explosion pressure to the external explosion pressure. It can be seen from this that the external explosion pressure can restrict the internal explosion pressure, and this constraint is coupled. This is consistent with the theoretical analysis. The increase in pressure and the extension of action time will increase the throwing speed of micro elements of the liner and affect the forming of the liner.

**Figure 6:**
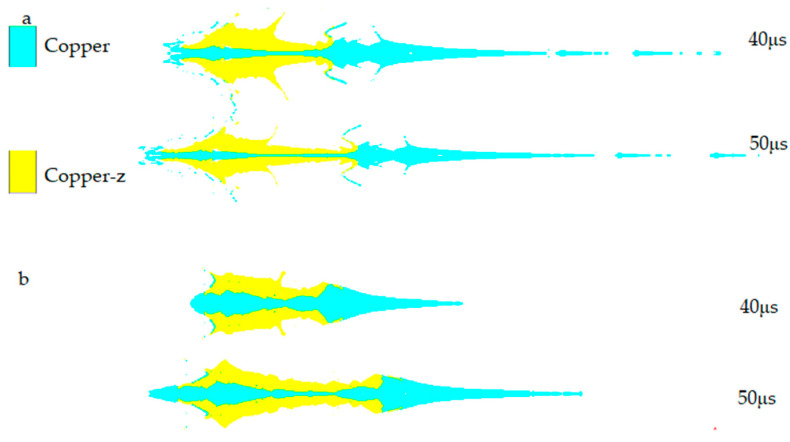
Jet shape: (**a**) composite charge; (**b**) integrated charging.

The shape after jet formation is shown in in Figure 6. In Figure 6a, the front part of the jet is mainly formed by a spherical missing body, and the truncated cone forms the middle part of the jet and the pestle. In Figure 6b, the head of the jet is formed by a spherical missing body, which is relatively short and thick, and the head diameter changes. Compared with Figure 6b, the composite charge significantly changes the shape of the jet and increases the length of the jet. At 40 μs, the length of the continuous section in Figure 6a is 63.16% higher than that in Figure 6b. According to the penetration theory of ideal incompressible hydrodynamics, under the same material, the penetration depth of the jet is directly proportional to the jet length. Therefore, the jet in Figure 6a is better than Figure 6b.

### 3.2. Effect of the Shell on the Jet Molding

**Figure 7:**
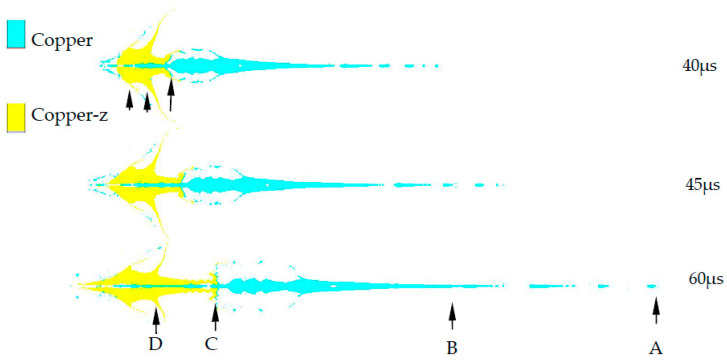
Jet morphology of charge without shell. “A, B, C, D” is the reference position.

**Figure 8:**
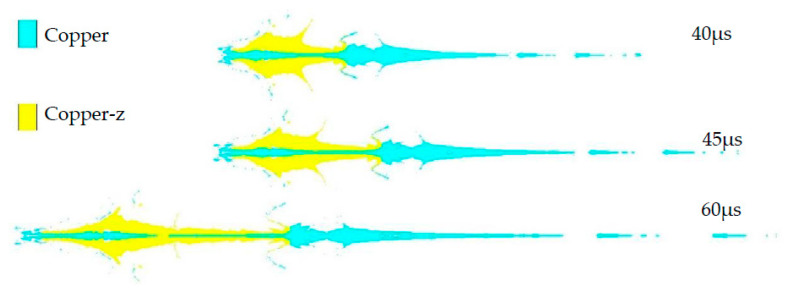
Jet morphology of charge with shell.

The SCS liner structure involved in this summary is shown in Figure 3. The influence of the charge shell on the jet structure is studied. “Shaped charge without shell” means that there is no shell “6” in Figure 1, and the constraint cylinder “3” in Figure 1 is polytetrafluoroethylene (PTFE). Because the strength of PTFE is far less than 45# steel, it can only play the role of positioning the internal and external charge columns. The jet formed by the shaped charge without shell is shown in Figure 7. The jet is obviously divided into two parts: the front and the rear. With the increase in time, the jet is elongated. Figure 8 shows the shape of the jet formed by the shaped charge with shell, and the two parts of the jet are more closely connected. Compared with Figure 7, “copper” still mainly forms the front of the jet, and “copper-z” forms the rear of the jet. It can be seen that the shell does not change the formation mode of the jet, but it can improve the continuity of the jet.

### 3.3. Effect of Wall Thickness of Confined Cylinder on Jet Formation

**Figure 9:**
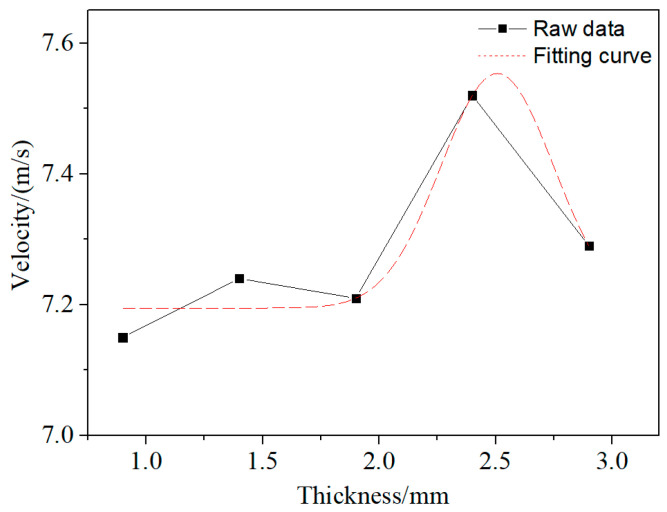
Variation curve of the continuous velocity of the explosive charge jet with the wall thickness of the constraint cylinder.

By changing the wall thickness of the constraint cylinder, the influence of the wall thickness of the constraint cylinder on the jet velocity is studied. With the increase in wall thickness of the constraint cylinder, the velocity of the continuous tip of the jet first increases and then decreases, as shown in Figure 9. The fitting results show that the continuous tip velocity of the jet approaches the maximum when the wall thickness of the constraint cylinder is 2.4 mm.

**Figure 10:**
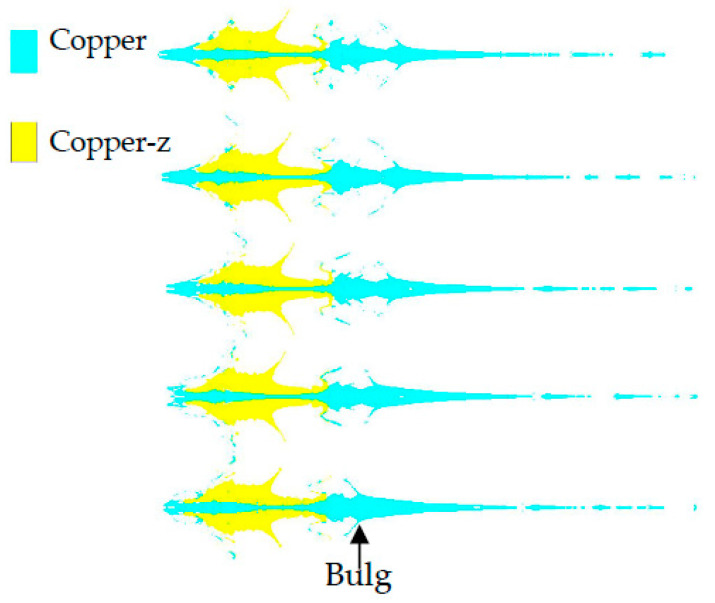
Jets formed by different wall thickness of confined cylinder.

Figure 10 shows the morphology of the jet when the constraint cylinder has different thicknesses. The wall thickness of the constraint cylinder corresponding to the jet is 0.9 mm to 2.9 mm from top to bottom. With the increase in the wall thickness of the constraint cylinder, the diameter of the jet protrusion decreases, and the part of the material in the spherical missing body forming the pestle body decreases.

### 3.4. Influence of the Top Wall Thickness of the Liner on Jet Formation

**Figure 11:**
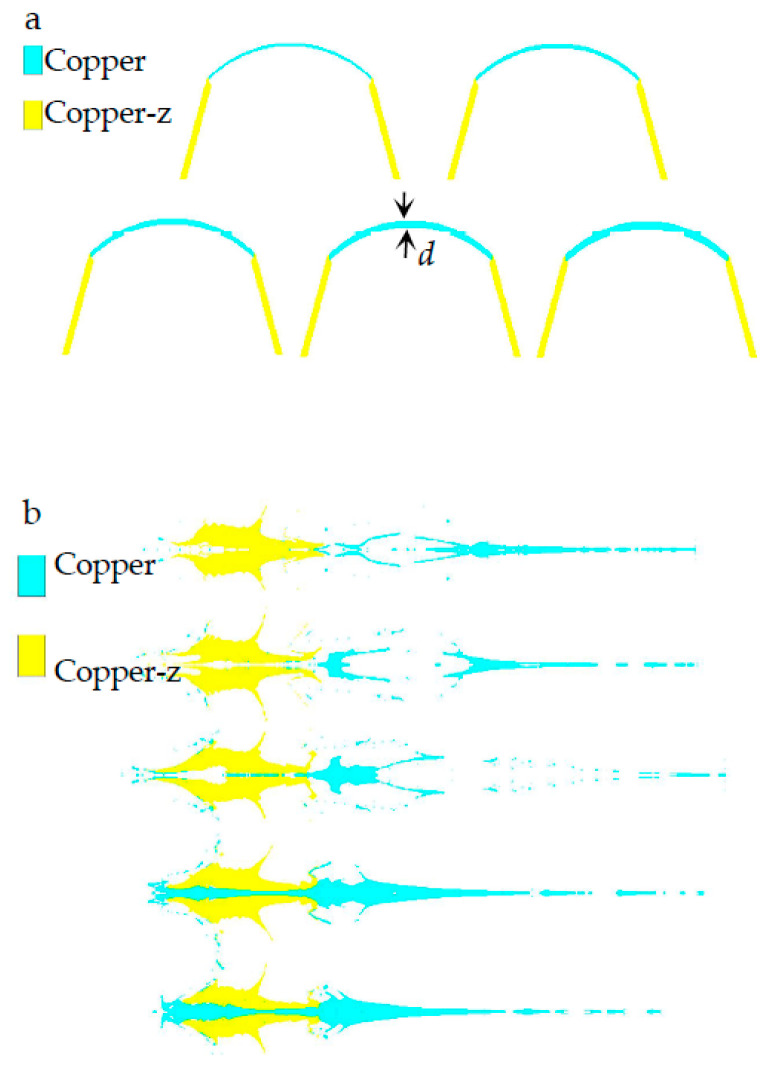
Jet shape formed by different vertex wall thicknesses: (**a**) SCS liner; (**b**) Jet.

The wall thickness of the liner directly affects the shape of the jet. On the premise of not changing the shape of the grain and the bus radius of the inner and outer surfaces of the spherical missing body, the influence of the top wall thickness of the spherical missing body on the jet shape is studied. The structure of the liner is shown in Figure 11a, and the wall thickness of the spherical missing body is measured by the top wall thickness (*d*). Figure 11b is the shape of the jet at 40 μs moment, which is *d* = 1~3 mm from left to right. With the increase in *d* value, the jet changes from divergence to continuity. When *d* = 1~2 mm, the jet head of the explosive charge fails to form. In Figure 11b, when *d* is from 2.5 mm to 3 mm, the length of the continuous section of the jet decreases. Therefore, it is meaningless to continue to increase the value of *d*. It can be seen that when *d* = 2.5 mm, the jet length and velocity of explosive charge are the best, and the pestle body is moderate.

### 3.5. Influence of Radius of Inner Circle Generatrix of Spherical Defect on Jet Formation

**Figure 12:**
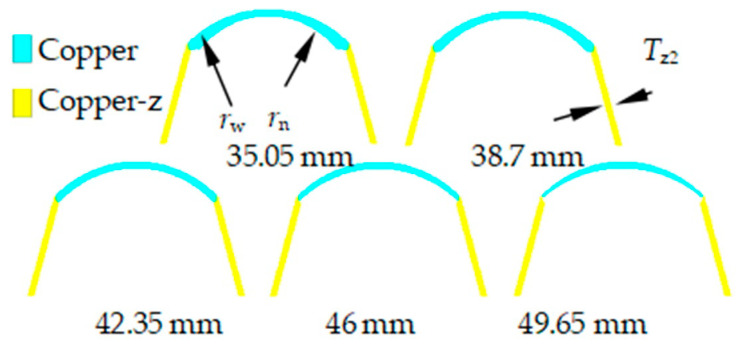
SCS liner with different inner surface bus radius.

According to the research results in Section 3.1, Section 3.2, Section 3.3 and Section 3.4, the wall thickness of the constraint cylinder is 2.4 mm, and the top wall thickness is 2.5 mm. Keep the radius *r*_w_ of the outer circular bus of the spherical missing body unchanged. Compare and study the influence of the radius *r*_n_ of the inner circular bus of the spherical missing body on the forming of the SCS liner. See Figure 12 for the relevant structures and parameters of the SCS liner. The radius of the inner circular bus of the spherical missing body is 35.05~49.35 mm, and the liner is shown in Figure 12.

**Figure 13:**
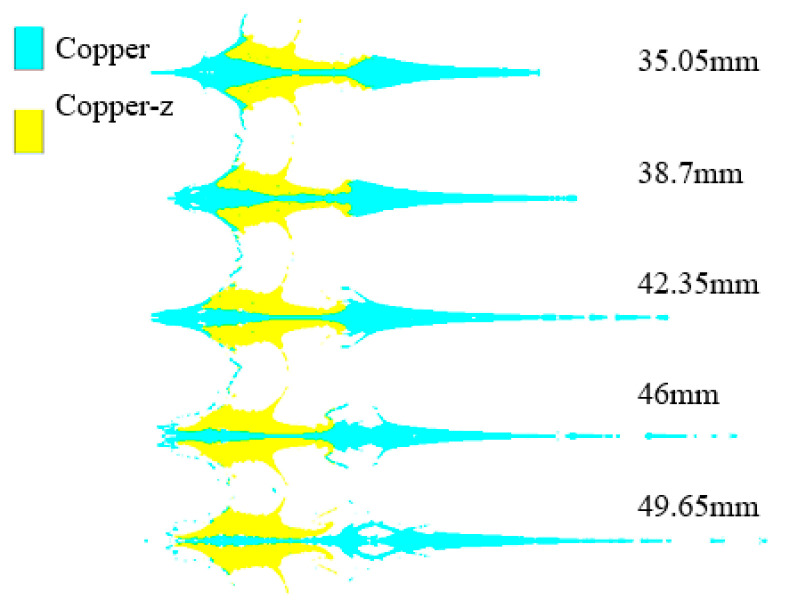
Jets formed by SCS liner with different inner surface bus radius.

Figure 13 shows the jet formed by SCS liners with different inner surface bus radiuses. With the increase in the inner surface bus radius, the jet length increases and the front diverges. When the inner surface bus radius is 35.05 mm and 38.7 mm, the jet is a continuous structure. When the inner circle bus radius is 38.7 mm, the jet length and continuity are the best. When the inner surface bus radius is 49.65 mm, the convex position is broken, and it is meaningless to continue to increase the inner surface bus radius.

### 3.6. Influence of Truncated Cone Shape on Jet Formation

**Figure 14:**
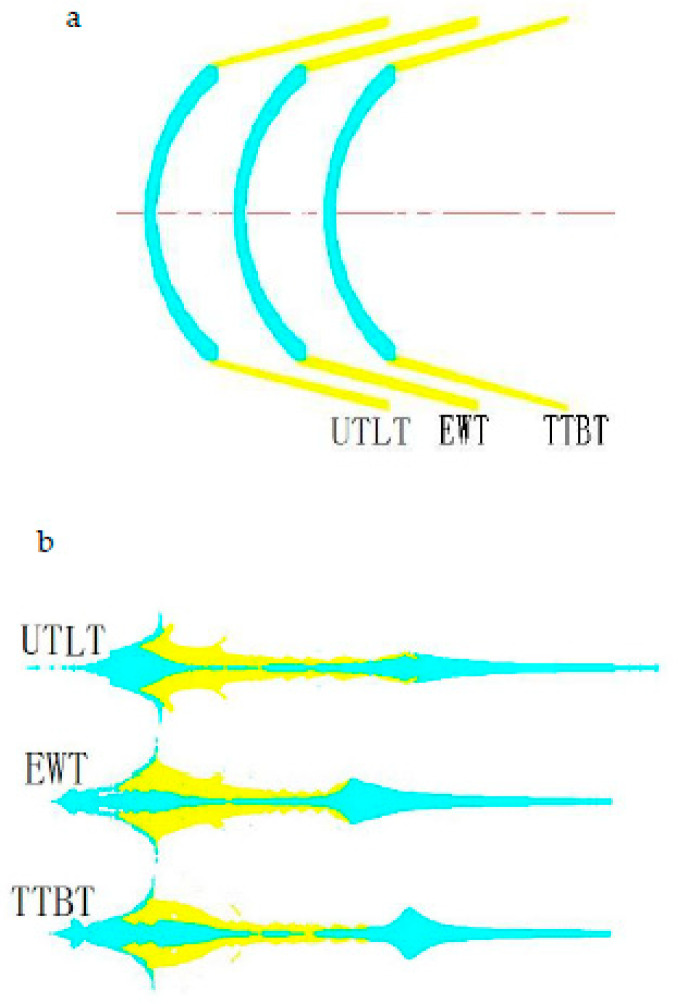
Jets formed by different truncated cones at the same time. (**a**) SCS liner with different truncated cones; (**b**) jet formed by SCS liner with different truncated cones.

As a part of the SCS liner, the truncated cone affects the forming of the SCS liner. According to the research conclusions in Section 3.1, Section 3.2, Section 3.3 and Section 3.4, the forming laws of different truncated cones are studied with a wall thickness of the constraint cylinder of 2.4 mm, a top wall thickness of the spherical missing body of *d* = 2.5 mm, an outer circular bus radius of 45.7 mm, and an inner circular bus radius of 38.7 mm, and the structure of the explosive column and shell remains unchanged. According to literature reports [4,5,15,16,17], cone-shaped charge liners generally include variable wall thickness (VWT) and equal wall thickness (EWT). Variable wall thickness can be divided into upper thin and lower thick (UTLT) and top thick and bottom thin (TTBT). Figure 14a is a SCS liner with different truncated cones, Figure 14b is the jet shape of the explosive charge. The UTLT jet in Figure 14b changes evenly, and there is a large bulge in the middle of the EWT and TTBT jets. Compared with the UTLT jet, the lengths of the EWT and TTBT jets are reduced by 11.43% and 10.71% respectively.

According to the theory of ideal incompressible jet and target penetration [18], the penetration depth is

(10) $$L=l\sqrt{\frac{\rho_{J}}{\rho_{T}}}$$

where *ρ_J_* is the density of the jet, *ρ_T_* is the density of the target, and *l* is the effective length of the jet. It can be seen that the penetration depth is directly proportional to the jet length.

**Figure 15:**
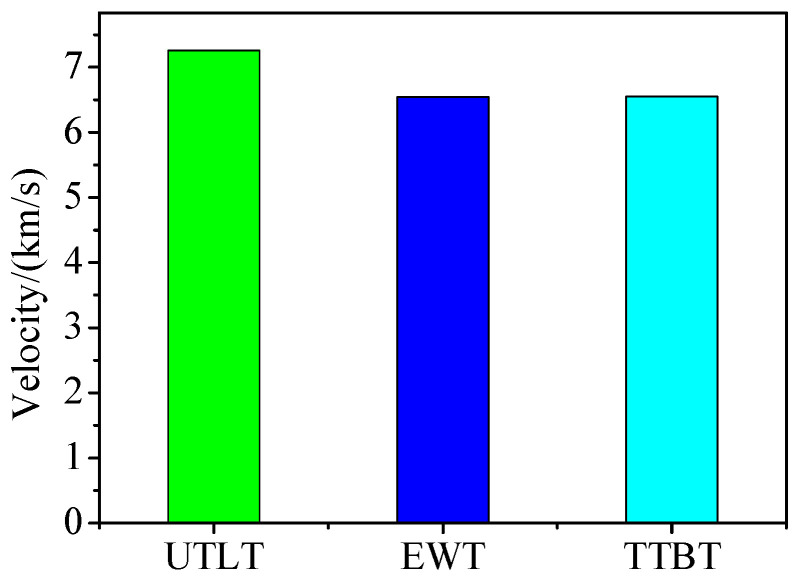
At 50 μs time head velocity of jet.

Figure 15 is a head velocity histogram of the explosive charge jet formed by different truncated cones. In the figure, the head velocities of UTLT are the highest, and the head velocities of EWT and TTBT are basically the same. Therefore, the explosive charge jet—after the SCS liner with a UTLT truncated cone is formed—has a uniform structure and high speed.

To sum up, the optimal scheme is: a composite charge with a constraint cylinder, a wall thickness of the constraint cylinder of 2.4 mm, a top thickness of the spherical segment charge liner of 2.5 mm, an outer circular bus radius of 45.7 mm, an inner circular bus radius of 38.7 mm, and a UTLT truncated cone.

### 3.7. Numerical Simulation of Jet Penetration

When the wall thickness of the constraint cylinder is 2.4 mm, the top thickness of the spherical missing body is 2.5 mm, the radius of the outer circle bus is 45.7 mm, and the radius of the inner circle bus is 38.7 mm, the numerical simulation study of jet penetration is carried out by using a UTLT truncated cone. The shaped jet is mapped into a Lagrangian mesh, and a 200 mm thick target plate model is established, as shown in Figure 10. We set fixed constraints around the target plate. The equation of state and parameters of copper, copper-z, and 45# steel are shown in [19], the strength model of copper and copper-z is Steinberg-Guinan, and the model parameters are shown in Table 2 [2]. The von Mises strength model is adopted for 45# steel, and the parameters are shown in Table 3. The process of the jet penetrating the target plate is simulated by the geometric failure model.

The von Mises strength model holds that when the equivalent stress at a point reaches a fixed value independent of the stress state, the material will yield, or the material is in the plastic state, and the equivalent stress is expressed with the principal stress as

(11) $${\left(\sigma_{1}-\sigma_{2}\right)}^{2}+{\left(\sigma_{2}-\sigma_{3}\right)}^{2}+{\left(\sigma_{3}-\sigma_{1}\right)}^{2}=2\sigma_{s}^{2}=6K^{2}$$

where *σ*_1_, *σ*_2_, *σ*_3_ are the principal stress in three mutually perpendicular directions at a certain point.

The Steinberg–Guinan strength model expresses the shear modulus and yield strength as functions of pressure, effective plastic strain, and internal energy (temperature), as follows

(12) $$G=G_{0}\left[1+(\frac{G_{p}^{\prime }}{G_{0}})\frac{p}{\eta^{1/3}}+(\frac{G_{T}^{\prime }}{G_{0}})(T-300)\right]$$

(13) $$Y=Y_{0}\left[1+(\frac{Y_{P}^{\prime }}{Y_{0}})\frac{p}{\eta^{1/3}}+(\frac{G_{T}^{\prime }}{G_{0}})(T-300)\right]\times {\left(1+\beta \varepsilon \right)}^{n}$$

where *ε* is the effective plastic strain, *η* = *v*_0_/*v* is the compressibility, *β* is the hardening constant, *n* is the hardening index, $G_{p}^{\prime }$ = *d*_G_/*d*_p_, $G_{T}^{\prime }$ = *d*_Y_/*d*_p_, $Y_{P}^{\prime }$ = *d*_Y_/*d*_p_, *Y*_0_ indicates that the condition which must be met is

(14) $$Y_{0}^{}{\left(1+\beta \varepsilon \right)}^{n}\leq Y_{\max}$$

where *Y*_max_ is the maximum yield strength.

**Figure 16:**
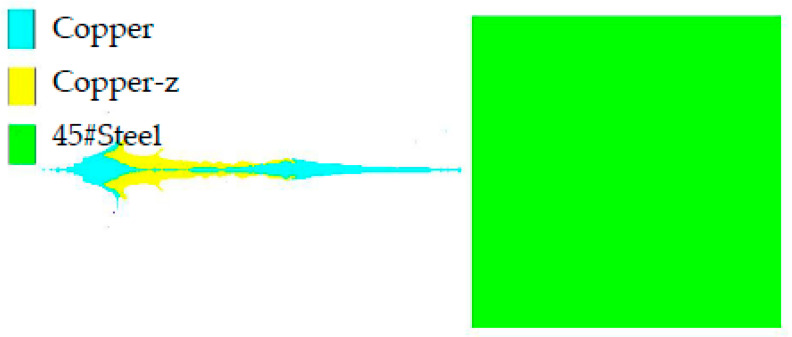
Penetration model of explosive charge jet.

**Figure 17:**
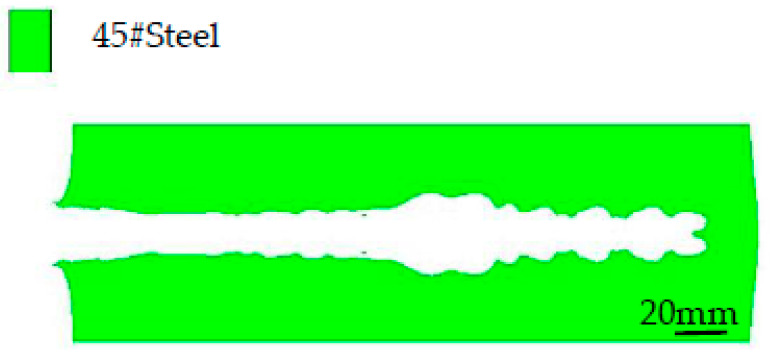
Numerical simulation of opening morphology.

The numerical simulation model of penetration is shown in Figure 16. The opening morphology of the target plate is shown in Figure 17. In Figure 17, the penetration depth is 185.00 mm, the diameter of the entry hole is 18.03 mm, and the maximum hole diameter is 24.94 mm. The perforation of the composite charge designed in this paper is straight and uniform, and the middle of the hole expands, which is conducive to the feeding of the follow-up materials, and can be referenced for the design of the series warhead [21].

## 4. The Forming and Penetration Test of the Spherical Cone Liner Based on the Explosive Pressure-Coupling Constraint Principle

### 4.1. X-ray Test of Jet Forming

**Figure 18:**
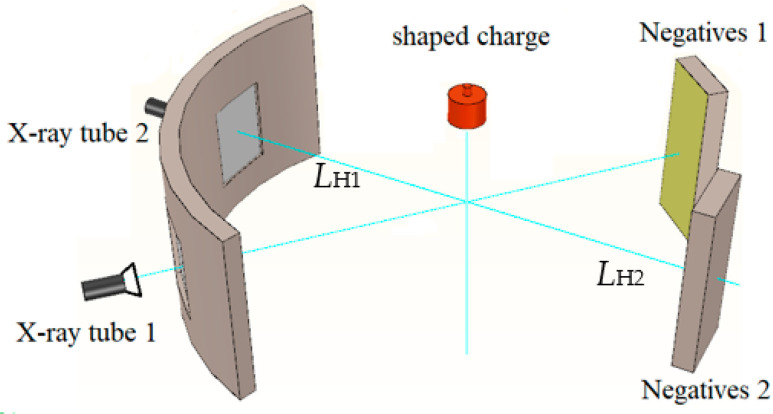
Field arrangement of pulse x-ray photography test and SCS liner.

The shaped charge in X-ray tests is a shell-free structure. The constraint cylinder is made of polytetrafluoroethylene, which is the same as the charge structure forming the jet in Figure 7; the SCS liner is shown in Figure 3, without a shell. The forming test of the SCS liner is photographed by two pulse X-ray machines (HP 450 KV), and the test layout is shown in Figure 18. The shaped charge is arranged vertically, with the two pulse X-ray machines placed at 45°, and the optical paths of the two pulse X-ray machines converged on the shaped charge axis, so that the jet passes through the intersection axis of the optical paths of the two pulse X-ray machines. The image on the X-ray photo is the enlarged image of the jet, and the magnification factor is determined by the relative positions of the pulse X-ray machine, shaped charge, and negative film.

**Figure 19:**
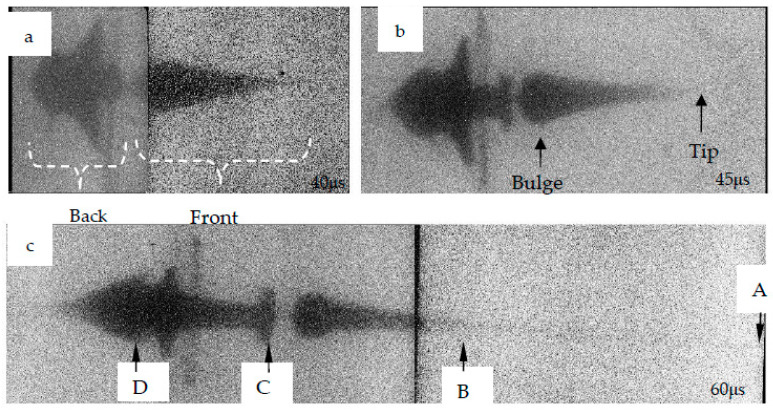
Jet image under pulsed X-ray: (**a**) 40 μs; (**b**) 45 μs; (**c**) 60 μs, “A, B, C, D” is the reference position.

In Figure 18, *L*_H1_ represents the distance from the light outlet of the pulse X-ray machine to the charging axis, and *L*_H2_ represents the distance from the charging axis to the negative film. Then, the amplification factor *K* can be obtained, *K* = *(L*_H1_ + *L*_H2)_/*L*_H1_. In the test, the exposure delay time of two pulse X-ray machines is set respectively. A metal wire of known length and diameter is placed in front of the negative box as a reference for the jet position. After the test, by measuring the distances from the corresponding positions of the jet on the two X-ray photos to the reference mark line, the movement distance of the corresponding position of the jet in the two exposure times can be calculated, and then the corresponding speed can be obtained.

(15) $$v_{0i}=\frac{L_{H2}-L_{H1}}{K\times \Delta t}$$

where *L*_*H*1_ is the distance from the corresponding position on the first photo to the reference mark line, *L*_*H*2_ is the distance from the corresponding position on the second photo to the reference mark line, Δ*t* is the exposure time difference, and *K* amplification factor. According to the X-ray photos, the length, diameter, head velocity, and tail velocity of the jet can be obtained, and the distribution positions of the spherical missing body and truncated cone materials in the SCS liner in the jet can also be obtained. According to the measurements in the test site, the amplification factor *K* is two, and the actual exposure time difference Δ*t* is 15 μs. The photographed jet morphology is shown in Figure 19, which is provided with reference mark lines.

Carry out speed measurement for the four positions (A, B, C, D) in Figure 7. See Table 4 for the measurement results. It is found from comparison that the error of numerical simulation is within a reasonable range. It can be seen that the numerical simulation is in good agreement with the X-ray tests, which proves that the numerical simulation of the SCS liner forming is reasonable.

Compared with the data provided in [2], the coaxiality of the jet in Figure 19 is better, and there is no obvious bending. The diameter of the jet is larger than that in [2], which is advantageous to increase the diameter of the hole on the steel plate penetrated by the jet.

### 4.2. Static Explosion Penetration Test

**Figure 20:**
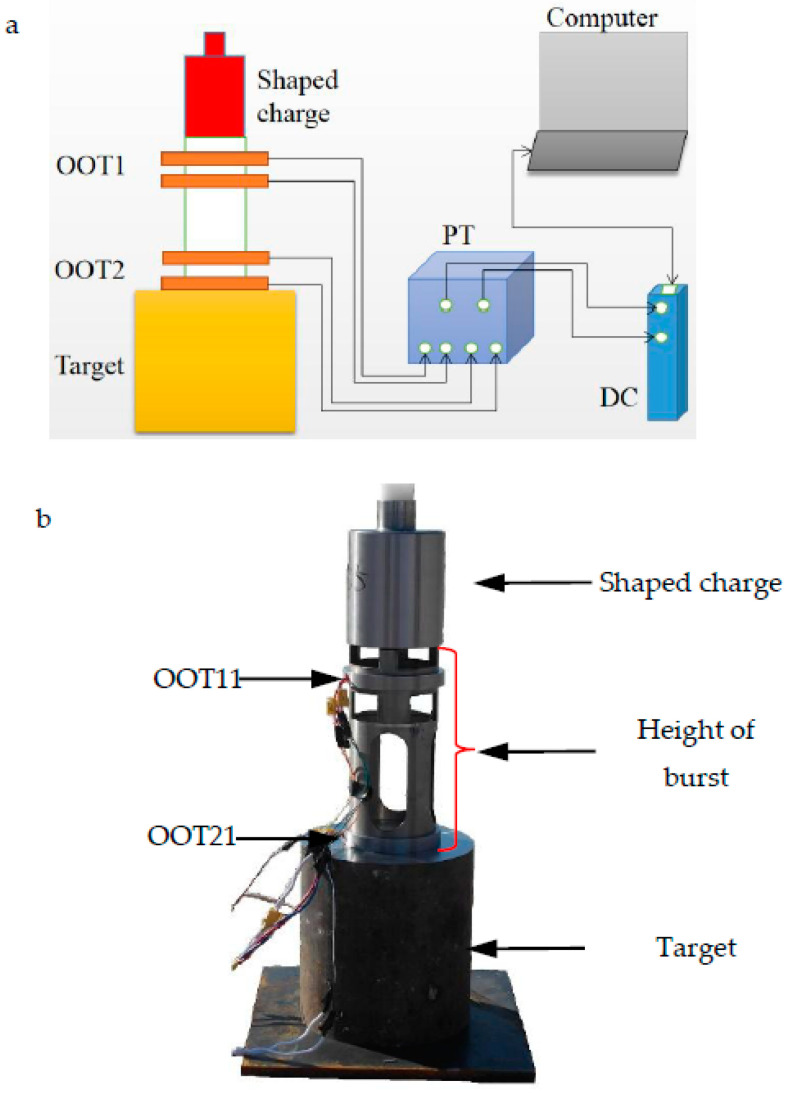
System composition diagram and experimental device: (**a**) speed measuring system; (**b**) test device.

We test and verify the numerical simulation in Section 3.7. The materials and structures of all components are the same as those of the numerical simulation. The measurement of the head velocity of the explosive charge jet. The system includes off-on target (Each off-on target is composed of two mutually isolated copper foils), wire, pulse trigger (PT for short, with a sensitivity of 0.1 µs), data collector (DC for short, 10 MHz), and computer. Refer to Figure 20. The explosive charge jet breaks through the off-on target (OOT for short) and turns on it, then the pulse trigger is excited to generate an electrical signal, and then the signal is recorded by the data collector. The time interval Δ*t* of the excitation signal of two off-on targets and the distance L between two off-on targets are measured, that is, the average velocity *v*_0_ of the explosive charge jet in this displacement is

(16) $$v_{0}=L/\Delta t$$

8701 explosive is pressed into the warhead and detonated by the center of the number eight electric detonator.

**Figure 21:**
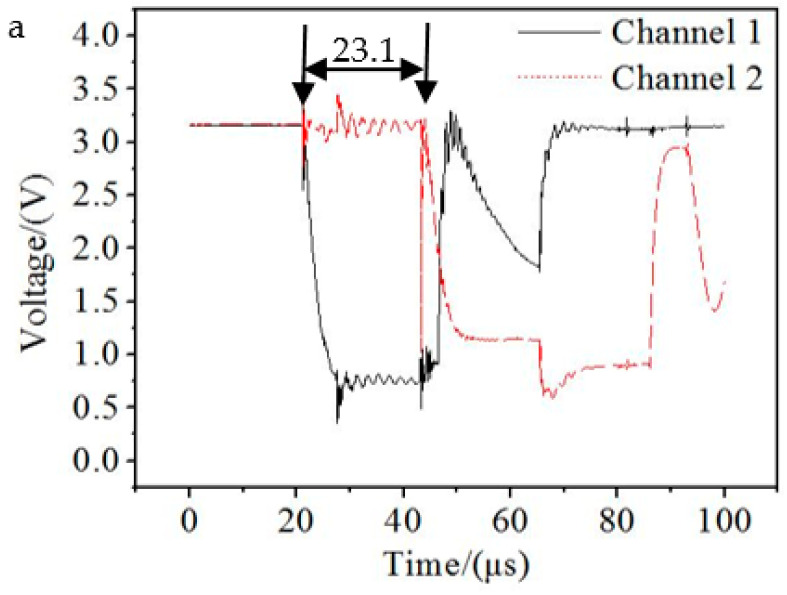

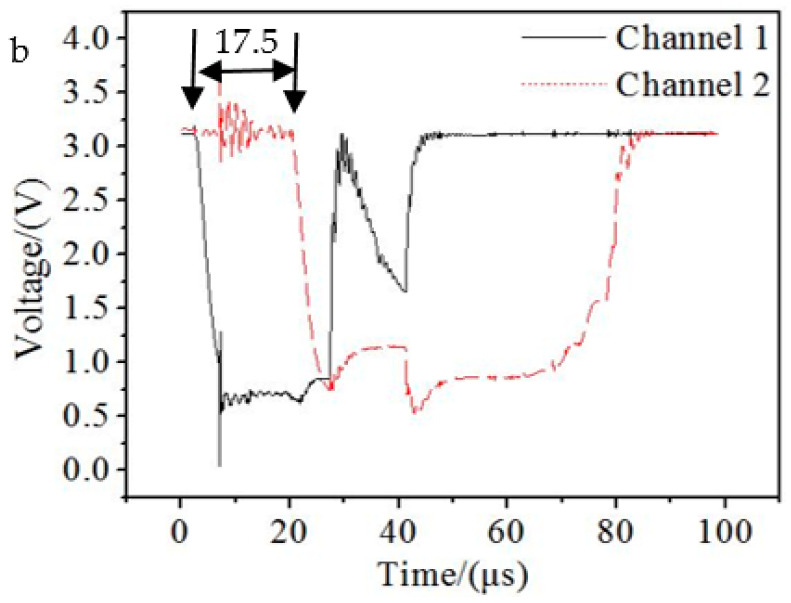
Pulse curve: (**a**) *L* = 174; (**b**) *L* = 174.

Two speed measurement tests were conducted to improve accuracy and repeatability. The distances *L* of the two tests are 174 mm and 134 mm respectively. The measured time is shown in Figure 21. According to Equation (16), the head velocities of the explosive charge jet are 7532.47 m/s and 7657.14 m/s respectively, and the average velocity is 7594.81 m/s. Compared with the average speed of the two tests in Figure 21, the head velocity error of the explosive charge jet in Figure 15 is 4.40%.

**Figure 22:**
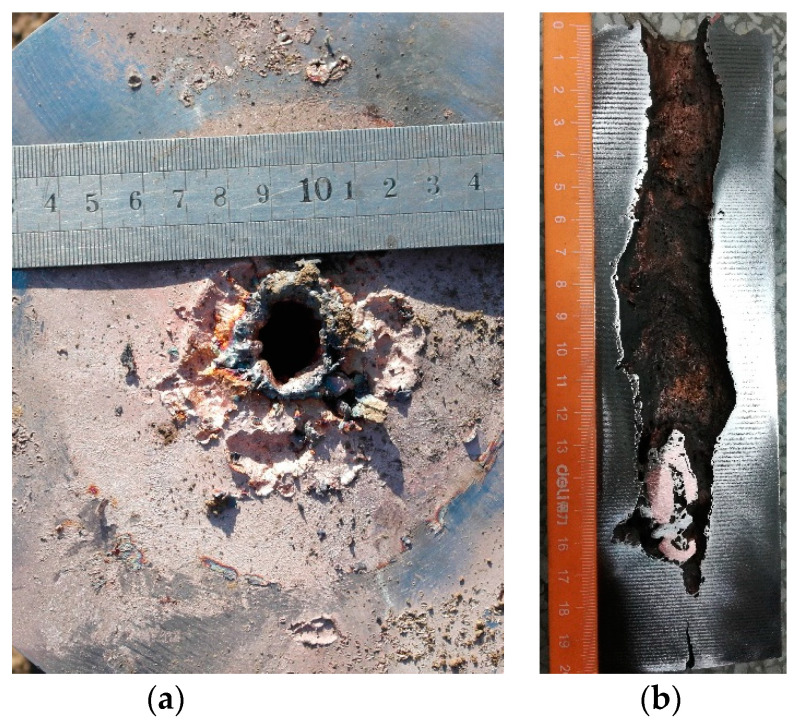
Target plate morphology: (**a**) into the hole; (**b**) perforation.

Figure 22 shows the target plate morphology after a static explosion test. The diameter of the entry hole is 20.35 mm and the penetration depth is 178.87 mm. As can be seen in Figure 22b, the deflection of the channel is caused by the radial velocity of the jet and the processing error of the liner [19]. The diameter of the entry hole is the smallest, and the middle increases to 32.37 mm, which is obviously better than the fine perforation and funnel-shaped perforation formed by the traditional charge structure. Maintaining the diameter of the perforation is a difficult problem, which is of great significance for damage and aftereffects [16]. The error of the numerical simulation is 11.30% in entry hole diameter and 3.43% in penetration depth. It can be seen that the numerical simulation is in good agreement with the experiment.

## 5. Conclusions

Herein, a new composite shaped charge based on the explosive pressure-coupling constraint principle is studied by means of numerical simulation and static explosion tests. The experiments verify that the theoretical analysis and numerical simulation are reasonable.

1. The charge structure based on the explosive pressure-coupling constraint principle increases the head velocity of the explosive pressure charge jet by 36.10% and the length of the continuous section by 32.51%.
2. The average head velocity of the explosive pressure charge jet in the test is 7594.81 m/s, and the error between this and the numerical simulation is 4.40%.
3. A shaped charge with a shell structure is more conducive to ensuring the continuity of the jet. When the thickness of the constraint cylinder is 0.9~2.4 mm, the head velocity of the jet increases with the increase in thickness. When the top wall thickness is 2.5 mm, the head velocity of the jet is high and the structure the best. Compared with the other two truncated cones, the explosive pressure charge jet formed by the UTLT truncated cone has a uniform structure and a higher velocity.

By using the split SCS liner and the explosion pressure-coupling constraint principle, the restriction of low speed after the forming of the spherical segment charge liner is broken through. The SCS liner can form a good follow-up jet, which provides a new idea for the development of liners. If the two structures of the liner are matched with different materials, the multi-functionality of the liner can be realized.

## Figures and Tables

**Table 1:** Equation of state parameters.

Material	Equation	*ρ* (g/cm^3^)	Gruneisen Coefficient	*C*_0_ (m/s)	*S_c_*	Temperature (K)
Copper	shock	8.900	2	3958	1.497	300
45#	shock	7.896	2.17	4569	1.49	300

**Table 2:** Material parameters (Steinberg–Guinan) [2].

Material	*Y* (MPa)	*Y*_max_ (MPa)	*Β* (None)	*N* (None)	*G*’_P_ (None)	*G*’_T_ (MPa)	*Y*’_P_ (None)	T_melt_ (K)
Copper	120.0	640.0	36.0	0.45	1.35	−17.98	0.003396	1790.0

**Table 3:** 45# steel parameters [20].

Material	Shear Modulus (GPa)	Yield Stress (MPa)
45# steel	79.4	355

**Table 4:** Comparison between jet velocity in numerical simulation and X-ray test results.

Name	Point A	Point B	Point C	Point D
X-ray (km/s)	11.02	4.92	2.49	1.03
Numerical simulation (km/s)	10.38	5.60	2.32	1.09
Error of numerical simulation (%)	5.81	13.82	6.83	5.83

## Data Availability

Not applicable.
